# Internet searches and heat-related emergency department visits in the United States

**DOI:** 10.1038/s41598-022-13168-3

**Published:** 2022-05-31

**Authors:** Quinn H. Adams, Yuantong Sun, Shengzhi Sun, Gregory A. Wellenius

**Affiliations:** 1 Department of Environmental Health, Boston University School of Public Health, Boston, MA USA; 2 Optum Labs Visiting Scholar, Eden Prairie, MN USA

**Keywords:** Environmental health, Climate-change adaptation

## Abstract

Emerging research suggests that internet search patterns may provide timely, actionable insights into adverse health impacts from, and behavioral responses to, days of extreme heat, but few studies have evaluated this hypothesis, and none have done so across the United States. We used two-stage distributed lag nonlinear models to quantify the interrelationships between daily maximum ambient temperature, internet search activity as measured by Google Trends, and heat-related emergency department (ED) visits among adults with commercial health insurance in 30 US metropolitan areas during the warm seasons (May to September) from 2016 to 2019. Maximum daily temperature was positively associated with internet searches relevant to heat, and searches were in turn positively associated with heat-related ED visits. Moreover, models combining internet search activity and temperature had better predictive ability for heat-related ED visits compared to models with temperature alone. These results suggest that internet search patterns may be useful as a leading indicator of heat-related illness or stress.

## Introduction

Heat is a recognized threat to public health as the links between high ambient temperatures and risk of both morbidity and mortality are well documented across the globe^1–3^. In the United States (US), despite continued increases in the availability of air conditioning, the burden of disease from days of extreme heat remains stubbornly high, with thousands of excess deaths attributable to heat each year^4,5^.

Across the US, the National Weather Service provides a heat early warning system to alert government agencies and the public in advance of days of extreme heat. A growing number of communities have implemented heat action plans that are activated in response to forecast days of extreme heat, and may include the opening of designated cooling centers, extended hours or reduced costs for public swimming pools, and timely public messaging about the health risks. The limited number of studies that have assessed the effectiveness of such early warning systems indicate that there is room for improvement of current heat early warning systems and resulting public health actions^6–9^.

Internet search patterns have been shown to be useful in tracking the incidence of influenza, dengue, and Lyme disease, among other communicable and noncommunicable diseases around the world^10–12^. Prior studies reveal that extreme weather events, including extreme heat, elicit an increase in information seeking via internet search^13^ and emerging research suggests that internet searches may provide novel insights into the population impacts of extreme heat. For example, in Shanghai, Li et al. (2016) showed that internet searches for the term “heat stroke” during the summer of 2013 were more strongly correlated with cases and deaths due to heat stroke as compared to maximum temperature^14^. Similarly, in England, Green et al. (2018) found that internet searches for terms related to heat or heat-related health symptoms were strongly correlated with signals from established local syndromic healthcare surveillance systems, especially calls to telehealth services^15^. Further, Jung et al. (2019) found that in Florida heat-related hospitalizations and emergency department (ED) visits were positively associated with Twitter messages related to air conditioning and heat as well as Google searches for “drink”, “heat stroke”, “park”, “swim”, and “tired”^16^. Young et al. (2021) observed an exponential increase in heat-related Twitter activity with increasing temperatures in the US, the United Kingdom, and Australia and concluded that social media data can provide important insight into the social response to heatwaves^17^. These prior studies suggest that internet search activity and/or social media posts may serve as a valuable and novel marker of the population-wide impacts of heat.

**Figure 1:**
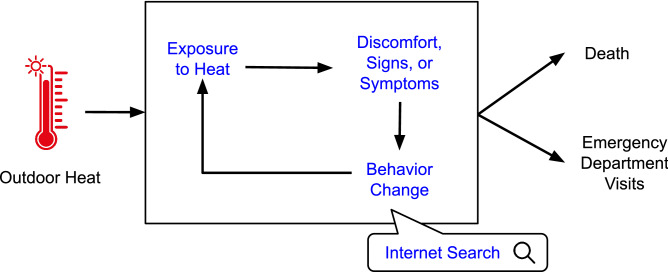
Conceptual framework highlighting the relationship between exposure to heat and health outcomes highlighting the additional, previously unmeasurable intermediate vulnerability that helps identify increased exposure, behavior change, and susceptibility measured using internet search activity.

We hypothesized that aggregated and anonymized data on internet search patterns can provide novel insights into the health effects or behavioral responses to extreme heat, as illustrated in the conceptual diagram shown in Fig. 1. Although there is ample evidence that days of extreme heat are linked to higher risk of death or healthcare utilization, there is typically little visibility into the experience of the much larger pool of people experiencing discomfort or preclinical or subclinical signs and symptoms of heat-related illness (typically unobserved variables are depicted in the square of Fig. 1). We posit that internet search activity can provide novel insights into these typically unobservable states.

If this conceptual model is correct, internet search patterns could provide a marker of the impact of heat on a given population that is available nearly everywhere and in near real-time. Specifically, internet searches may serve as a marker of risk perception (e.g., searches for dehydration or heat stroke) or intent to change behavior (e.g., searches for swimming pools or air conditioning), and thus, may provide important insight into periods of time when or where populations are experiencing acute concern about heat. To test this hypothesis, we linked aggregated and anonymized data on internet search patterns provided by Google Trends with weather data and ED visit data for heat-related illnesses among adults across the US with commercial health insurance.

## Methods

### Google search data

Daily data on internet search activity was extracted using the Google Health Trends Application Programming Interface (API)^18^ for 30 United States Nielson designated market areas (DMA). A DMA region is a group of counties that make up a geographic area commonly used to define television and radio markets. We retrieved daily queries during the warm season (May through September) from 2016 through 2019 associated with common heat-related health symptoms as well as terms related to known protective actions against heat stress including air conditioning and swimming pools^19^. We considered a number of possible heat-related search terms selected a priori based on existing literature. Selections were then narrowed down based on search frequencies in our study regions during the study time period. Search terms with only zero values over the study period were excluded. The final list of quoted search strings included “heat”, “heat stroke”, “dehydration”, “heat rash”, “heat exhaustion”, “swimming pool”, and “air conditioning”. In addition to standard search strings, we retrieved available knowledge graph entities using freebase identifier codes to obtain all searches that were related to each topic including typos and indirect descriptions (Supplementary Table S1). The Google Health Trends API returns the proportion of search sessions executed in a given place on a given date that satisfies the corresponding search term restriction. A total of seven quoted search strings were used in this analysis with the addition of four corresponding freebase identifiers for heat, dehydration, swimming pool, and air conditioning collectively referred to as “search terms” hereafter.

### Emergency department (ED) visit data

We obtained de-identified administrative claims during the warm season (May to September) between 2016 and 2019 from the OptumLabs Data Warehouse (OLDW), which includes medical and pharmacy claims, laboratory results, and enrollment records for commercially insured and Medicare Advantage enrollees^20^. The database contains longitudinal health information on enrollees and patients, representing a diverse mixture of ages, ethnicities, and geographical regions across the US. We identified claims for ED visits based on the International Classification of Diseases (ICD) code, revenue code, Current Procedural Terminology (CPT) code, and place of service code. For each claim, we then extracted information on the age, sex, and county of residence of the individual, as well as the admission date and principal diagnosis code (based on ICD-10). We grouped ED visits for heat-related illness (ICD-10: T67, E86, E87, X30) by DMA areas^21^. We limited our analysis to ED visits occurring among individuals aged 18 and older. This study involved analysis of pre-existing, de-identified data and was exempted from institutional review board approval.

### Heat assessment

Because heat stress is experienced differently across individuals and communities, a single metric of heat is unlikely to be optimal in every population, location, or circumstance. Ambient temperature has been shown to be associated with heat-related morbidity and is a commonly used marker of thermal discomfort in the epidemiological literature^22^, but heat index (HI) and wet bulb globe temperature (WBGT) may provide a more accurate physiological representation of thermal discomfort or heat stress. Specifically, HI, which is frequently used as the basis for issuing heat warnings and advisories in the US, is a combination of air temperature and relative humidity and provides a representation of how heat feels to the human body^23^. WBGT accounts for air temperature, relative humidity, wind speed, and solar radiation and is thus thought to better depict heat stress in direct sunlight^24^. Given that no single meteorological index may fully represent the population experience of heat stress, we assessed three possible metrics of heat.

Daily maximum ambient temperature, HI, and WBGT were derived using data from the National Land Data Assimilation System Phase 2 (NLDAS-2) forcing dataset^25^. The NLDAS-2 data are available at a spatial resolution of approximately 12 km across the contiguous United States and each metric was aggregated to the county level for the analysis. Daily maximum HI was calculated following the National Weather Service algorithm using the *weathermetrics* R package^23^ with air temperature, specific humidity, and atmospheric pressure variables from the NLDAS-2 dataset. We estimated daily maximum WBGT as a function of ambient temperature, relative humidity, incident solar radiation, wind speed, and atmospheric pressure, following the algorithm of Liljegren et al.^26^.

To characterize population exposure to heat, we calculated population-weighted means of daily maximum ambient temperature, HI, and WBGT for each day in each of the 30 DMAs using population counts per county from the 2010 US Census. Specifically, we multiplied daily ambient temperature, HI, and WBGT separately by the proportion of the total DMA population within each county and summed the results to obtain a daily time series with the population weighted maximum ambient temperature, HI, and WBGT for each day and for each DMA. The results for ambient temperature are presented in the main text and the results from analyses of HI and WBGT are available in the supplement.

## Statistical analysis

The statistical analysis was divided into three parts: (1) assessment of the association between temperature and internet search activity, (2) assessment of the association between internet search activity and heat-related ED visits, and (3) evaluation of a series of models for predicting heat-related ED visits based on combinations of internet search activity and weather metrics.

### Association between heat and Google search activity

We first estimated the Spearman correlation coefficients between daily maximum temperature and each of the selected heat-related Google searches. Next, we used a two-stage approach to assess the relationship between maximum temperature and Google search activity across the 30 DMAs^27,28^. In the first stage, we fit a quasi-Poisson regression combined with a distributed lag non-linear model (DLNM) separately in each DMA to estimate the DMA-specific association between daily maximum temperature and Google search activity, adjusting for day of week (categorical), federal holiday, year (categorical), and month (categorical). We estimated exposure–response relationships using a quadratic B-spline with an internal knot at the 50th percentile of each DMA-specific temperature distribution^29^. We modelled the lag-response function using a natural cubic B-spline with two internal knots placed at equal intervals on the log scale, with the maximum lag up to 5 days.

In the second stage, we used a multivariate random effects meta-analytic model to estimate the overall cumulative association between maximum daily temperature and heat-related internet searches across the 30 DMAs. We report the exponentiated coefficient derived from this model as the ratio of the proportion of searches for extreme heat at the 95th versus 1st percentile of the DMA-specific temperature distribution, loosely referred to throughout as the relative risk (RR). We repeated these analyses using maximum WBGT and HI instead of temperature, with similar results (see supplement).

### Association between Google search activity and heat-related ED visits

We next examined the association between Google search activity and heat-related ED visits in each DMA by conducting an analogous two-stage analysis as described above, but with Google searches as the exposure of interest rather than daily maximum temperature. We continued to adjust for the same time varying factors described above. We report the RR for heat-related ED visits for the 95th versus 1st percentile of DMA-specific Google search activity.

### Evaluation of model performance for prediction of heat-related ED visits

We then evaluated whether Google search activity, alone or in combination with meteorologic variables, could be used as a predictor of heat-related ED visits. To examine this hypothesis, we fit a series of models using the distributed lag nonlinear model framework described above. We fit models including either a non-linear lagged effect of maximum daily temperature, a non-linear lagged effect of searches for specific terms, or both. All models were adjusted for federal holidays, year, month, and day of week. The non-linear exposure-lag-response function for maximum daily temperature was modeled as in the analyses described above. For internet searches, we modeled the exposure–response function using a quadratic B spline with one knot placed at the 50^th^ percentile of DMA-specific distribution of Google search activity and modeled the lag-response function using a natural cubic B spline with two knots placed at equal intervals on the log scale of lags up to five days. We evaluated the performance of each model using the sum of quasi-AIC (lower is better), the median pseudo-R^2^ (higher is better), and the median of the mean square errors (lower is better) across DMA-specific models.

We performed sensitivity analysis where we replaced daily maximum temperature with the other two heat metrics: daily maximum HI and WBGT in all models to test the robustness of our prediction for ED visits.

All analyses were conducted in R (version 4.0.2), using software packages *dlnm* version 2.4.6 and *mvmeta* version 1.0.3.

## Results

**Figure 2:**
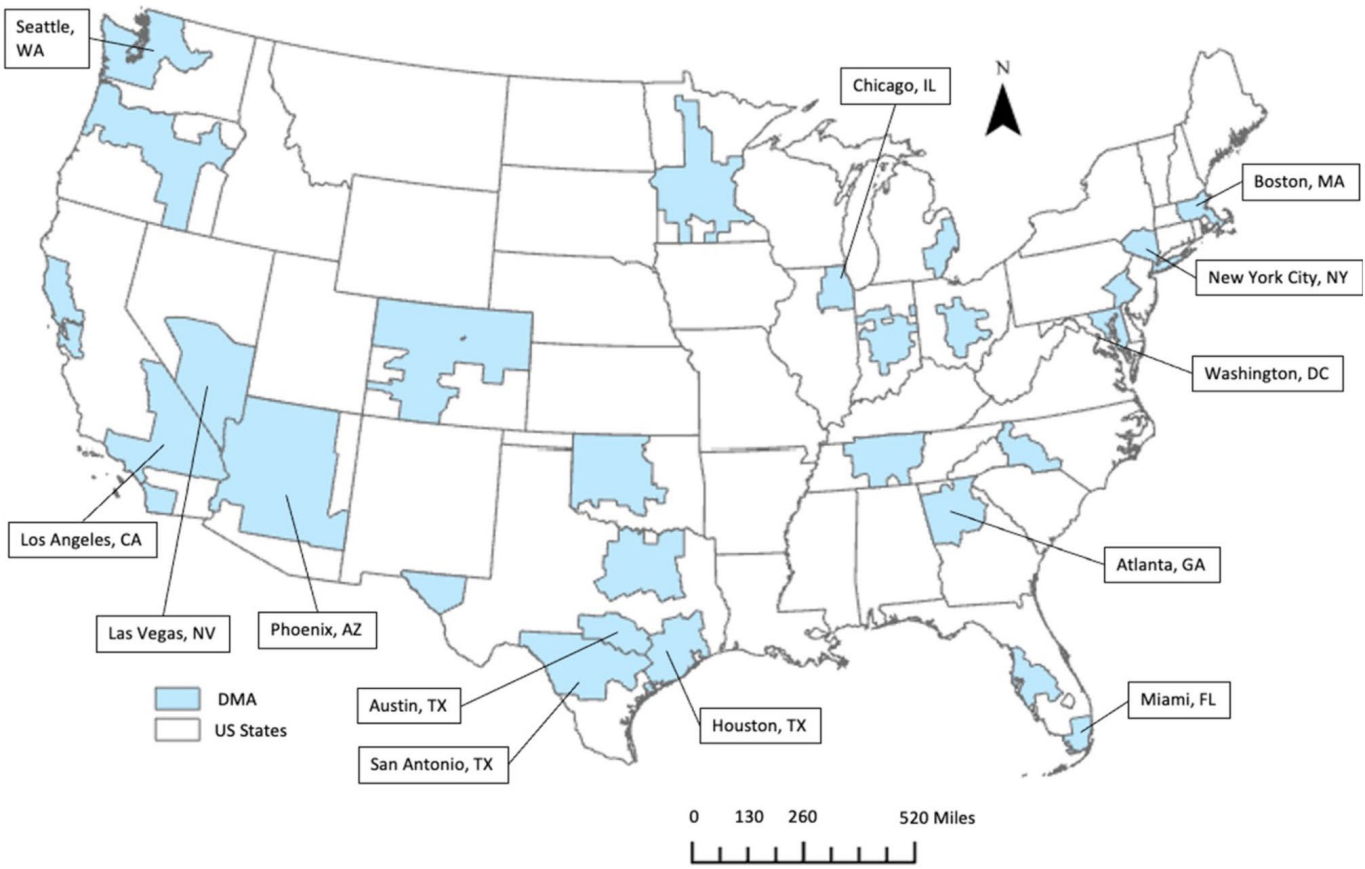
Location of the 30 Nielsen Designated Marketing Areas (DMA) defined by one or more US counties for which Google Trends and ED data was available. County and state shapefiles provided by the United States Census Bureau TIGER/Line shapefiles. Map created using ArcGIS Pro version 2.8 (Esri).

We obtained estimates of the proportion of daily Google searches for a given search term or topic using the Google Health Trends API^18^. Daily estimates were available for each of the 30 Nielson designated market areas (DMA) across the US. DMAs generally approximate US metropolitan areas, and include urban centers, suburban areas and the surrounding counties that receive the same television signals (Fig. 2). Our analysis is limited to the warm seasons (May through September) of 2016 through 2019. We considered both quoted search strings (e.g., “swimming pool”) and the corresponding search topic or knowledge graph entity as denoted by a freebase identifier (FBID)^30^. Note that not all search strings have a corresponding FBID (Supplementary Table S1).

**Figure 3:**
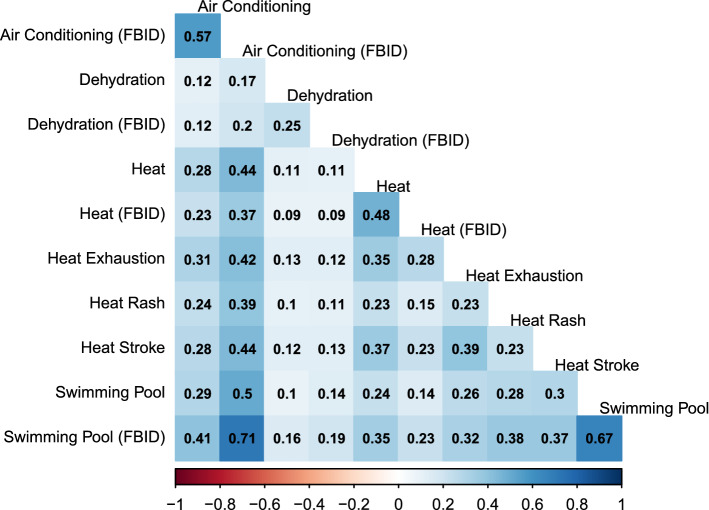
Median Spearman Correlation coefficients between Google search terms among 30 DMAs.

We first examined the correlations among search volumes for our pre-selected list of terms and topics. Searches for specific terms and their corresponding FBID-defined topic tended to be strongly correlated (Fig. 3). Beyond that, the strongest correlations were observed between searches for air conditioning (as an FBID topic) and swimming pool as an FBID topic (r = 0.71), and quoted search strings for swimming pool (r = 0.50), heat (r = 0.44), heat stroke (r = 0.44), and heat exhaustion (r = 0.42). Other correlations among search terms were more modest (r < 0.40).

**Figure 4:**
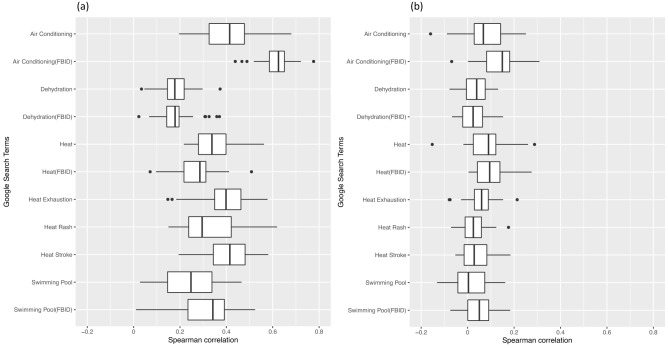
Spearman correlation distribution of (**a**) daily maximum ambient temperature and same day heat-health related Google searches and (**b**) heat-health related Google searches and same day heat-related ED visits across 30 DMAs.

We next evaluated the correlations between daily maximum ambient temperature and Google searches on the same day within each DMA (Fig. 4a). Maximum daily temperature tended to be most strongly correlated with searches for air conditioning (FBID), although the strength of the correlation varied considerably across DMAs. Google searches were also associated with ED visits for heat-related illness on the same day among adults with commercial health insurance (Fig. 4b). Searches for air conditioning (FBID) tended to be most strongly correlated with ED visits, but with notable heterogeneity among DMAs.

**Figure 5:**
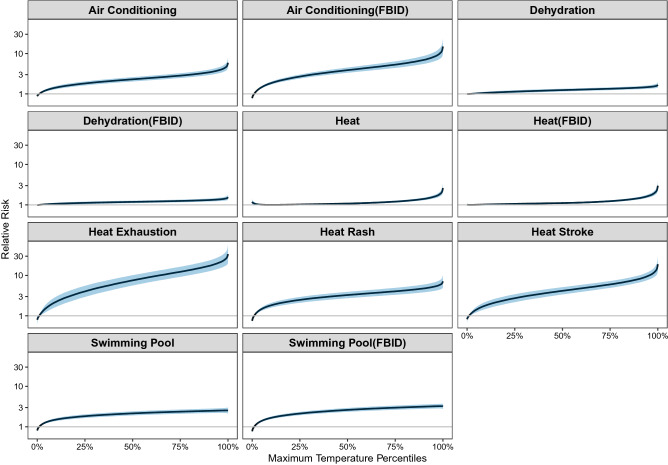
Cumulative exposure–response curve (95% CI shaded in light blue) for the relative risk for Google searches related to maximum ambient temperature percentile over lags 0–5 days. Relative risk on the y-axis is displayed on the log scale. Reference temperature: 1st percentile of DMA-specific daily maximum ambient temperature distribution.

We next used distributed lag nonlinear models to assess the association between percentiles of maximum daily ambient temperature and Google searches for selected terms, adjusting for temporal trends (Fig. 5). Ambient temperature was positively associated with the probability of Google searches for the selected terms. The association was most pronounced for searches for heat exhaustion (RR: 20.5; 95% CI: 14.7, 28.4) comparing the 95th to the 1st percentile of the DMA-specific distribution of warm-season maximum daily temperature); followed by searches for heat stroke (RR: 9.8; 95% CI: 7.8, 12.2); and searches for air conditioning using the FBID (RR: 8.2; 95% CI: 6.3, 10.6) (Supplementary Table S2). In other words, internet searches for air conditioning and heat stroke were nearly 10-times more common on the hottest versus the coolest days, while searches for heat exhaustion were approximately 20-times more common.

The associations between temperature and the volume of Google searches were most pronounced on the same day (lag 0) or one day after a day of extreme heat (lag 1), but with some evidence of the association persisting for an additional 1–2 days for searches for heat exhaustion and heat stroke (Supplementary Fig. S1).

**Figure 6:**
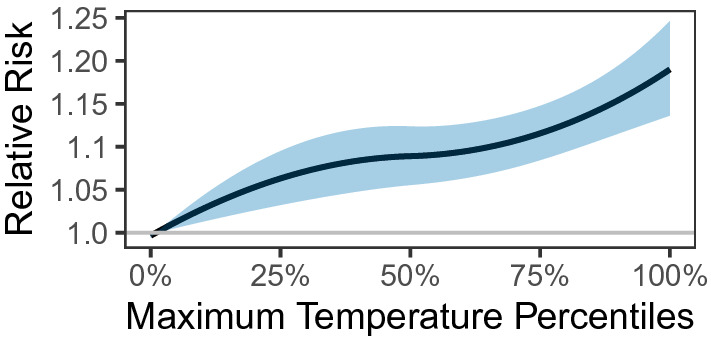
Cumulative exposure–response curve for the relative risk of heat-related ED visits for percentiles of DMA-specific daily maximum ambient temperature over lags 0–5 days. Reference search volume: 1st percentile of DMA-specific daily maximum ambient temperature distribution.

We used the same approach to estimate the relationship between DMA-specific daily maximum temperature percentile and ED visits for heat-related illness (Fig. 6) Warm-season temperatures were associated with monotonically higher risk of ED visits. For example, daily maximum temperature was associated with 1.17 times (95% CI: 1.13, 1.22) the risk of ED visits for heat-related illness, comparing the DMA-specific 95th percentile of maximum temperature to the 1st percentile. This relationship is most pronounced for same day temperature and ED visits (lag 0) (Supplementary Fig. S2).

**Figure 7:**
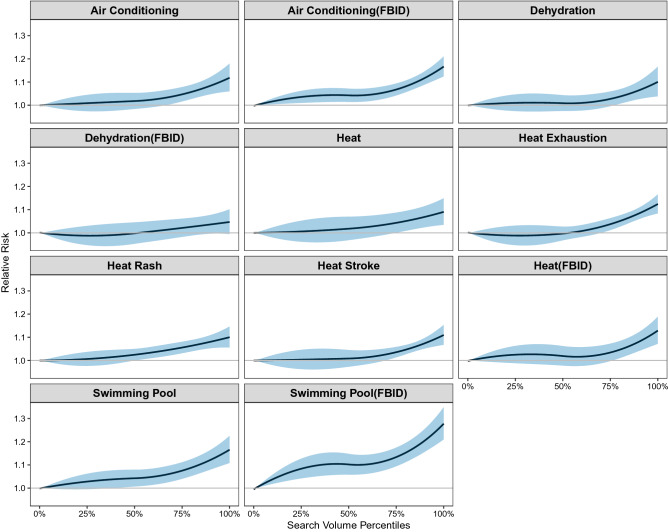
Cumulative exposure–response curve for the relative risk of heat-related ED visits for percentiles of DMA-specific search terms over lags 0–5 days. Reference search volume: 1st percentile of DMA-specific search volume distribution.

Search volumes for the selected Google search terms were each associated with a higher relative risk for ED visits (Fig. 7). The strongest association was observed for searches for swimming pools (FBID) (RR: 1.24, 95% CI: 1.18, 1.30) and air conditioning (FBID) (RR: 1.14, 95% CI: 1.11, 1.18). These associations were most pronounced on the same day (lag 0), but with some evidence that searches on a given day are associated with ED visits on the next day (lag 1) (Supplementary Fig. S3).

Finally, we fit a series of models to assess the relative abilities of temperature alone (Model 1), Google search activity alone (Model 2), or both together (Model 3) to predict heat-related ED visits. We assessed model fit using both pseudo R^2^ (higher is better) and mean squared error (MSE; lower is better) (Table 1). The median pseudo R^2^ was consistently highest for models that included both temperature and search terms (Model 3) and was lowest for the model with temperature only (Model 1). Results were qualitatively similar when considering the MSE (Table 1) or the sum of quasi-AIC (Supplementary Table S3) rather than the pseudo-R^2^, suggesting that models that included both temperature and search terms tended to perform better in predicting heat-related ED visits versus models with temperature or search terms alone. For example, comparing Model 1 and Model 3, we found that the pseudo R^2^ increased by 11.4% on average across 11 different Google searches, while mean square error decreased by 2.3%.

**Table 1:** Model comparison with median pseudo-R square^1^ (mean square error^2^).

Search terms:	Temperature only (Model 1)	Search terms only (Model 2)	Temperature + search terms (Model 3)
–	0.210 (0.0939)	–	–
Heat	–	0.226 (0.0926)	0.235 (0.0920)
Heat stroke	–	0.227 (0.0923)	0.233 (0.0915)
Heat exhaustion	–	0.234 (0.0924)	0.241 (0.0917)
Swimming pool	–	0.215 (0.0926)	0.229 (0.0918)
Air conditioning	–	0.224 (0.0923)	0.231 (0.0917)
Dehydration	–	0.223 (0.0930)	0.234 (0.0920)
Heat rash	–	0.222 (0.0925)	0.235 (0.0917)
Heat (FBID)	–	0.221 (0.0924)	0.235 (0.0917)
Swimming pool (FBID)	–	0.235 (0.0919)	0.236 (0.0914)
Air conditioning (FBID)	–	0.233 (0.0921)	0.235 (0.0917)
Dehydration (FBID)	–	0.215 (0.0928)	0.229 (0.0917)

*FBID* freebase identifier.

^1^The median of pseudo-R squared across 30 DMAs.

^2^The mean of mean square error across 30 DMAs.

### Alternative heat metrics

Estimates of WBGT, HI, and ambient temperature were highly correlated, and results were qualitatively similar regardless of heat metric considered. Specifically, HI was highly correlated with both WBGT (r = 0.95) and ambient temperature (r = 0.94), while WBGT and ambient temperature were somewhat less strongly correlated (r = 0.85). Results of all analyses repeated using WBGT or HI rather than ambient temperature are shown in supplementary Figs. 4–11 and supplementary Tables 3–6.

## Discussion

Across 30 US metropolitan areas, using historical data on weather, internet search patterns, and ED visits among insured individuals, we found that (1) daily maximum temperature was associated with substantially more Google searches for selected terms, and (2) that Google searches are associated with ED visits for heat-related illnesses. Moreover, including the search volume of specific Google searches improved the model fit for the prediction of heat-related ED visits compared to a model based only on temperature and accounting for temporal trends. These results suggest that internet search patterns may provide novel predictive insights, above and beyond those already available from meteorological variables, that may be useful for near real-time assessment of the adverse health impacts of heat and plausibly inform future heat early warning and response systems. Our findings are consistent with the conceptual model shown in Fig. 1 and suggests that internet searches can serve as a marker and provide additional information regarding people’s experience with, or response to, warm-season temperatures.

Heat early warning systems are a key component in the public health response to extreme heat. However, heat warnings are issued for large geographic areas, with little opportunity to tailor the alerts to specific populations or communities. Moreover, the effectiveness of early warning systems in reducing heat-related morbidity and mortality remains suboptimal^6,31–33^.The results of this study raise the intriguing possibility that, in the future, heat early warning systems may be able to incorporate and leverage data about the population impacts of extreme heat available in near real-time, in addition to forecast and observed weather conditions. Such a system would blur the line between a heat early warning and a heat health surveillance system with the potential to alert community leaders and public health practitioners about evolving conditions in real time. The additional information that captures how the population is being affected, behaviorally and physically, to heat can also help supplement current public health surveillance, especially in parts of the world where traditional public health surveillance is lacking.

A large and growing literature indicates that warm-season temperatures are associated with increasing risk of human discomfort or illness, manifesting as increased risk of death or healthcare utilization^3,34^. We posit that search can serve as a marker of people’s concerns over their health (e.g., searches for symptoms of heat exhaustion or heat stroke), as well as their intention or desire to reduce their exposure to heat (e.g., searches for swimming pools and air conditioning). Indeed, there are numerous examples in the literature that demonstrate the utility of internet search patterns for monitoring or predicting communicable^10–12^ and noncommunicable disease outcomes^35,36^. Our findings are also consistent with those of prior heat-related studies in Shanghai, England, and Florida^14–16^ showing that internet search patterns and other passively collected signals (e.g., from social media) can provide timely and novel insights into the impacts of extreme heat on population health and wellbeing. Our analyses extend this prior work by applying distributed lag non-linear models to data from 30 populous metropolitan areas across the US and linking search patterns, three separate metrics of heat, and rates of ED visits for heat-related illness at a population level.

Our findings reveal that internet searches provide information about heat health impacts above and beyond ambient temperature. This is evident by comparing Figs. 6 and 7 and noting that six out of the eleven selected search terms were more strongly associated with ED visits than was temperature. Similar to the findings from Li et al. (2016)^14^, we found that internet searches for specific search terms or topics better predict heat-related ED visits than temperature alone. Moreover, models including both ambient temperature and internet searches were better able to predict heat-related ED visits compared to either metric alone.

The timing of the observed associations is notable. Specifically, heat was most strongly associated with heat-relevant searches on the same day. Interestingly, we found an apparent peak in the association between ambient temperature and searches for heat rash, heat exhaustion, and heat stroke approximately one to two days following a day of extreme heat. This result may reflect an increase in searches by individuals who sought medical care and later used the internet to seek more information about their diagnosis or symptoms, although this remains speculative. Similarly, internet search activity was also most strongly associated with heat-related ED visits on the same day, but with some residual association evident the following day. This pattern of results is consistent with search as a marker of acute discomfort in response to warm weather.

Overall, searches for heat exhaustion, swimming pools, and air conditioning appeared to be most indicative of heat impacts, given their strong association with both maximum daily temperature and heat-related ED visits. These search terms also yielded the predictive models with the highest pseudo R^2^ and lowest MSE. This pattern of results suggests that search activity may reflect a combination of population concern for the adverse health effects of heat and adaptive behaviors intended to minimize these impacts.

These results need to be considered in light of several important potential limitations. First, there is no information available regarding the demographic makeup of Google users, and it is possible that the most vulnerable individuals are less well represented in these aggregate data. For example, the elderly, young children, or people in low-income communities may be at the highest risk for heat-related illness and may also be less likely to be active internet users compared to more affluent populations between the ages of 18 and 64^37,38^. Second, the aggregation of search data across a large geographic area also precludes evaluation of how search behavior varies across communities within a metropolitan area. Indeed, our current analyses are based on the DMA, which are typically larger than metropolitan areas and include multiple US counties. If more granular data were available in the future, it might be possible to examine how impacts of and responses to heat differ by neighborhood. Such data could plausibly be used to develop more geographically specific heat response plans. Third, it is unknown how stable the observed relationships might be over time. Any public health action based on aggregated search activity would need to be regularly re-evaluated and optimized. Finally, we only had data on ED visits among those with commercial health insurance, potentially excluding individuals without health insurance that may be particularly susceptible to the health impacts of heat.

## Conclusions

In conclusion, notwithstanding important limitations, this study demonstrates that aggregated search activity is associated with daily maximum temperature, heat index, and WBGT as well as ED visits for heat-related illnesses and suggests that search activity can provide novel insights into population health that may be useful for risk mitigation in the context of extreme heat. Additional studies are needed to understand how or whether such signals can be usefully integrated into public health practice or aid in public health preparedness and response.

## Supplementary Information

Supplementary Information.
