# Supplementary material for: Internet searches and heat-related emergency department visits in the United States
Source: Sci Rep. 2022 May 31;12:9031. doi: 10.1038/s41598-022-13168-3 (PMC9156736; doi:10.1038/s41598-022-13168-3)
Supplement: Supplementary file 1 — Supplementary Information. [file 41598_2022_13168_MOESM1_ESM.docx]

**Supplementary Materials**

**Internet Searches and Heat-Related Emergency Department Visits in the United States**

Quinn H. Adams^1^, Yuantong Sun^1^, Shengzhi Sun^1,2^, Gregory A. Wellenius^1,2^

^1^Department of Environmental Health, Boston University School of Public Health, Boston, Massachusetts

^2^OptumLabs Visiting Scholar, Eden Prairie, MN, United States of America

***Table S1****: Complete list of Search Terms and Corresponding Freebase Identifier Codes*

| **Search Terms:** | **WikiData**  **Search Code** | **Corresponding**  **Freebase Identifier** |
| --- | --- | --- |
| **Heat** | [Q69032838](https://www.wikidata.org/wiki/Q44432) | [/m/03k2v](https://freebase.toolforge.org/m/03k2v) |
| **Heat Stroke** | [Q69032838](https://www.wikidata.org/wiki/Q69032838) | NA |
| **Dehydration** | [Q194290](https://www.wikidata.org/wiki/Q194290) | [/m/014961](https://freebase.toolforge.org/m/014961) |
| **Heat Rash** | N/A | N/A |
| **Heat Exhaustion** | [Q12643772](https://www.wikidata.org/wiki/Q12643772) | N/A |
| **Swimming Pool** | [Q1501](https://www.wikidata.org/wiki/Q1501) | [/m/0b_rs](https://freebase.toolforge.org/m/0b_rs) |
| **Air Conditioning** | [Q173725](https://www.wikidata.org/wiki/Q173725) | [/m/025wky1](https://freebase.toolforge.org/m/025wky1) |

***Figure S1:*** *Time course for the relative risk of heat-related Google searches and 95% confidence interval for extreme high daily maximum temperature (95^th^ percentile of DMA-specific daily maximum temperature) with reference to the 1^st^ percentile of DMA-specific daily maximum temperature over lag 0-5 days. Note that the relative risk is displayed on the log scale.*

***Figure S2:*** *Time course for the relative risk of heat-related ED visits and 95% confidence interval for extreme high daily maximum temperature (95^th^ percentile of DMA-specific daily maximum temperature) with reference to the 1^st^ percentile of DMA-specific daily maximum temperature over lag 0-5 days.*


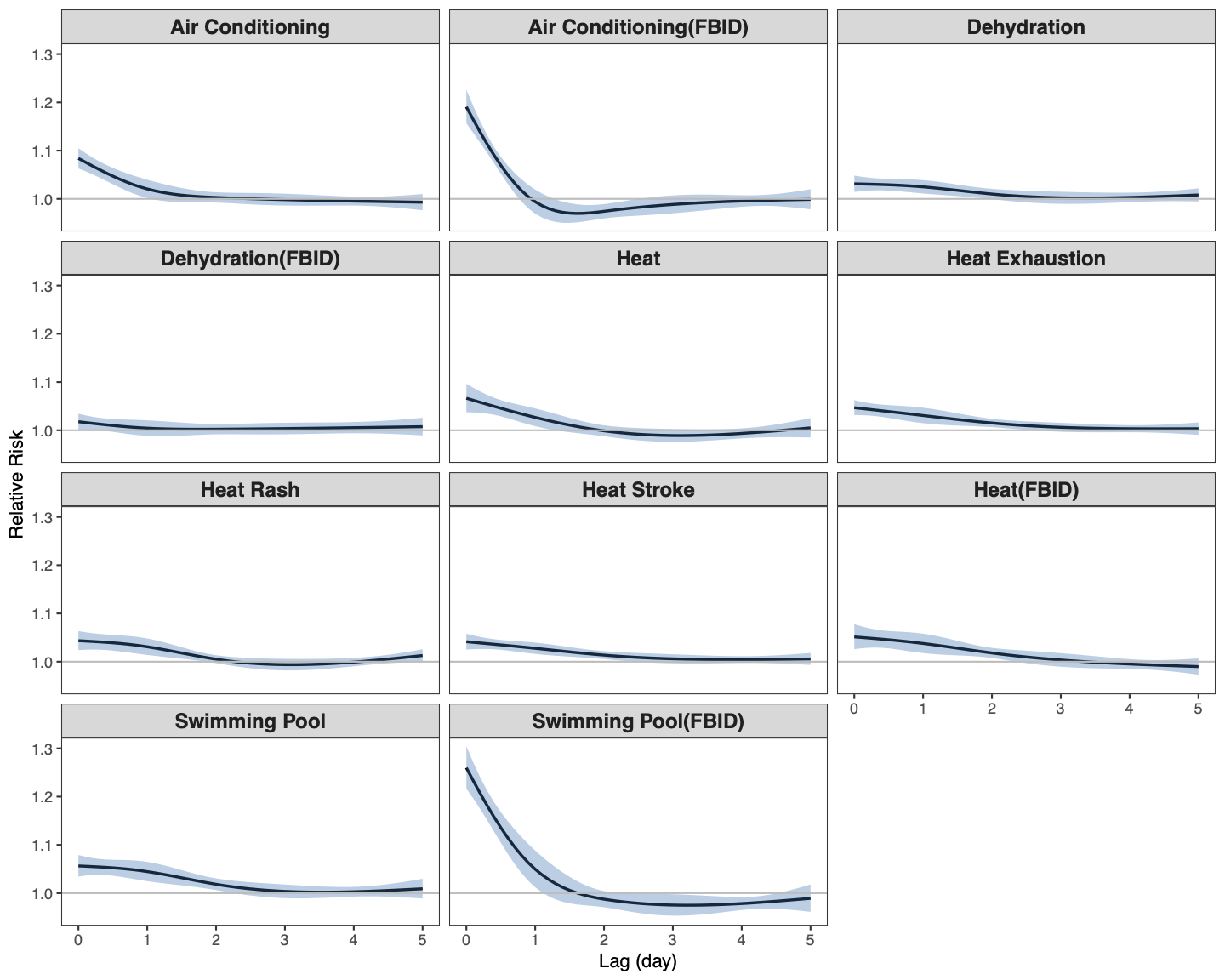


***Figure S3:*** *Time course for the relative risk of heat-related ED visits and 95% confidence interval for extreme high Google search volume (95^th^ percentile of DMA-specific search volume distribution) with reference to the 1^st^ percentile of DMA-specific search volume distribution over lag 0-5 days.*

***Table S2:*** *Relative Risk (95% confidence interval) for associations between daily maximum temperature and searches at the 95th and 97.5th percentile of DMA-specific maximum temperature; searches and ED visits at the 95th and 97.5th percentile of DMA-specific search volume; and maximum temperature and ED visits at the 95th and 97.5th percentile of DMA-specific maximum temperature. RRs are estimated with reference to the first percentile of searches or ED visits.*

| **Search Terms:** | **Temperature and Searches (95th percentile)** | **Temperature and Searches (97.5th percentile)** | **Searches and ED visits (95th percentile)** | **Searches and ED visits (97.5th percentile)** | **Temperature and ED visits (95^th^ percentile** | **Temperature and ED visits (97.5th percentile)** |
| --- | --- | --- | --- | --- | --- | --- |
| -- | -- | -- | -- | -- | 1.17 (1.13,1.22) | 1.18 (1.13,1.23) |
| **Air Conditioning** | 3.784 (3.24, 4.54) | 4.93 (3.95, 6.15) | 1.10 (1.05,1.15) | 1.11 (1.06,1.16) | -- | -- |
| **Air Conditioning (FBID)** | 8.19 (6.34, 10.59) | 11.65 (8.26, 16.43) | 1.14 (1.11,1.18) | 1.15 (1.12,1.19) | -- | -- |
| **Dehydration** | 1.48 (1.35, 1.63) | 1.59 (1.41, 1.81) | 1.08 (1.03,1.13) | 1.09 (1.04,1.15) | -- | -- |
| **Dehydration (FBID)** | 1.36 (1.24, 1.49) | 1.47 (1.30, 1.65) | 1.04 (1.00,1.09) | 1.04 (1.00,1.09) | -- | -- |
| **Heat** | 1.57 (1.43, 1.72) | 2.09 (1.78, 2.45) | 1.08 (1.03,1.13) | 1.08 (1.03,1.14) | -- | -- |
| **Heat (FBID)** | 1.66 (1.51, 1.82) | 2.29 (1.96, 2.68) | 1.10 (1.06,1.15) | 1.12 (1.06,1.17) | -- | -- |
| **Heat Exhaustion** | 20.46 (14.72, 28.42) | 28.09 (18.48, 42.70) | 1.10 (1.07,1.13) | 1.11 (1.08,1.15) | -- | -- |
| **Heat Rash** | 5.23 (4.12, 6.65) | 6.31 (4.71, 8.46) | 1.09 (1.06,1.13) | 1.10 (1.06,1.14) | -- | -- |
| **Heat Stroke** | 9.78 (7.82, 12.23) | 14.46 (10.56, 19.80) | 1.09 (1.06,1.12) | 1.10 (1.06,1.14) | -- | -- |
| **Swimming Pool** | 2.52 (2.21, 2.87) | 2.54 (2.18, 2.95) | 1.14 (1.09,1.19) | 1.15 (1.10,1.21) | -- | -- |
| **Swimming Pool (FBID)** | 3.21 (2.84, 3.63) | 3.24 (2.83, 3.71) | 1.24 (1.18,1.30) | 1.26 (1.20,1.32) | -- | -- |

***Table S3:*** *Model comparison using the sum of quasi-AIC*

| **Search Terms:** | **Temperature Only** | **WBGT/HI Only** | **Search Terms Only** | **Temperature + Search Terms** | **WBGT + Search Terms** | **HI + Search Terms** |
| --- | --- | --- | --- | --- | --- | --- |
| **--** | 104925.5 | 104983.6/  104920.6 | -- | -- | -- |  |
| **Heat** | -- | -- | 104521.4 | 104315.2 | 104349.5 | 104323.7 |
| **Heat Stroke** | -- | -- | 104537.8 | 104292.0 | 104337.7 | 104292.6 |
| **Heat Exhaustion** | -- | -- | 104549.8 | 104339.4 | 104388.4 | 104341.4 |
| **Swimming Pool** | -- | -- | 104585.8 | 104360.6 | 104404.1 | 104357.2 |
| **Air Conditioning** | -- | -- | 104517.9 | 104315.9 | 104366.9 | 104320.2 |
| **Dehydration** | -- | -- | 104710.7 | 104405.2 | 104458.5 | 104403.6 |
| **Heat Rash** | -- | -- | 104624.6 | 104359.7 | 104418.4 | 104366.2 |
| **Head (FBID)** | -- | -- | 104529.5 | 104313.9 | 104367.3 | 104332.3 |
| **Swimming Pool (FBID)** | -- | -- | 104228.5 | 104108.9 | 104134.4 | 104096.5 |
| **Air Conditioning (FBID)** | -- | -- | 104348.7 | 104267.6 | 104302.0 | 104264.6 |
| **Dehydration (FBID)** | -- | -- | 104672.5 | 104337.3 | 104403.3 | 104341.0 |

***Table S4:*** *Model comparison with median pseudo-R square^1^ (mean square error^2^)*

| **Search Terms:** | **Max Temp/WBGT/HI Only (Model 1)** | **Search Terms Only**  **(Model 2)** | **Temperature + Search Terms (Model 3)** | **WBGT + Search Terms**  **(Model 3)** | **HI+ Search terms**  **(Model 3)** |
| --- | --- | --- | --- | --- | --- |
| **--** | 0.210 (0.0939)  0.209 (0.0939)/  0.209(0.0938) | -- | -- | -- |  |
| **Heat** | -- | 0.226 (0.0926) | 0.235 (0.0920) | 0.233 (0.0920) | 0.231 (0.0919) |
| **Heat Stroke** | -- | 0.227 (0.0923) | 0.233 (0.0915) | 0.229 (0.0916) | 0.232 (0.0915) |
| **Heat Exhaustion** | -- | 0.234 (0.0924) | 0.241 (0.0917) | 0.236 (0.0917) | 0.239 (0.0916) |
| **Swimming Pool** | -- | 0.215 (0.0926) | 0.229 (0.0918) | 0.227 (0.0919) | 0.228 (0.0918) |
| **Air Conditioning** | -- | 0.224 (0.0923) | 0.231 (0.0917) | 0.227 (0.0917) | 0.229 (0.0917) |
| **Dehydration** | -- | 0.223 (0.0930) | 0.234 (0.0920) | 0.228 (0.0921) | 0.232 (0.0920) |
| **Heat Rash** | -- | 0.222 (0.0925) | 0.235 (0.0917) | 0.230 (0.0917) | 0.232 (0.0916) |
| **Heat (FBID)** | -- | 0.221 (0.0924) | 0.235 (0.0917) | 0.233 (0.0918) | 0.231 (0.0917) |
| **Swimming Pool (FBID)** | -- | 0.235 (0.0919) | 0.236 (0.0914) | 0.237 (0.0914) | 0.236 (0.0913) |
| **Air Conditioning (FBID)** | -- | 0.233 (0.0921) | 0.235 (0.0917) | 0.233 (0.0918) | 0.235 (0.0917) |
| **Dehydration (FBID)** | -- | 0.215 (0.0928) | 0.229 (0.0917) | 0.228 (0.0918) | 0.229 (0.0916) |

Abbreviations: FBID = freebase identifier

^1^the median of pseudo-R squared across 30 DMA areas

^2^the mean of mean square error across 30 DMA areas

**Assessment of Wet Bulb Globe Temperature:**

***Figure S4:*** *Cumulative exposure-response curve for the relative risk for Google searches related to maximum WBGT percentile over lags 0-5 days. Relative risk on the y-axis is displayed on the log scale. Reference temperature: 1st percentile of DMA-specific daily maximum WBGT distribution.*

***Figure S5:*** *Time course for the relative risk of heat-related Google searches and 95% confidence interval for extreme high daily maximum WBGT (95^th^ percentile of DMA-specific daily maximum WBGT) with reference to the 1^st^ percentile of DMA-specific daily maximum WBGT over lag 0-5 days. Note that the relative risk is displayed on the log scale.*

**

***Figure S6:*** *Cumulative exposure-response curve for the relative risk of heat-related ED visits for percentiles of DMA-specific daily maximum WBGT over lags 0-5 days. Reference search volume: 1^st^ percentile of DMA-specific daily maximum WBGT distribution.*

**

***Figure S7:*** *Time course for the relative risk of heat-related ED visits and 95% confidence interval for extreme high daily maximum WBGT (95^th^ percentile of DMA-specific daily maximum WBGT) with reference to the 1^st^ percentile of DMA-specific daily maximum WBGT over lag 0-5 days.*

***Table S5:*** *Relative Risk (95% confidence interval) for associations between daily maximum WBGT and searches at the 95th and 97.5th percentile of DMA-specific maximum WBGT; and WBGT and ED visits at the 95th and 97.5th percentile of DMA-specific maximum WBGT. RRs are estimated with reference to the first percentile of searches or ED visits.*

| **Search Terms:** | **WBGT and Searches (95th percentile)** | **WBGT and Searches (97.5th percentile)** | **WBGT and ED visits (95^th^ percentile** | **WBGT and ED visits (97.5th percentile)** |
| --- | --- | --- | --- | --- |
| -- | -- | -- | 1.16 (1.11,1.21) | 1.17 (1.12,1.22) |
| **Air Conditioning** | 3.66 (3.16, 4.24) | 4.75 (3.87, 5.83) | -- | -- |
| **Air Conditioning (FBID)** | 7.35 (5.91, 9.15) | 10.53 (7.83, 14.14) | -- | -- |
| **Dehydration** | 1.41 (1.31, 1.53) | 1.48 (1.33, 1.66) | -- | -- |
| **Dehydration (FBID)** | 1.36 (1.25, 1.48) | 1.49 (1.33, 1.67) | -- | -- |
| **Heat** | 1.61 (1.51, 1.73) | 2.15 (1.84, 2.52) | -- | -- |
| **Heat (FBID)** | 1.59 (1.49, 1.71) | 2.19 (1.91, 2.50) | -- | -- |
| **Heat Exhaustion** | 17.35 (12.22, 24.63) | 26.33 (16.66, 41.59) | -- | -- |
| **Heat Rash** | 5.20 (4.12, 6.57) | 6.60 (4.97, 8.77) | -- | -- |
| **Heat Stroke** | 8.22 (6.42, 10.52) | 12.56 (8.89, 17.74) | -- | -- |
| **Swimming Pool** | 2.48 (2.15, 2.87) | 2.65 (2.22, 3.16) | -- | -- |
| **Swimming Pool (FBID)** | 3.12 (2.67, 3.62) | 3.29 (2.74, 3.94) | -- | -- |

**Assessment of Heat Index:**

******

***Figure S8:*** *Cumulative exposure-response curve (95% CI shaded in light blue) for the relative risk for Google searches related to maximum heat index percentile over lags 0-5 days. Relative risk on the y-axis is displayed on the log scale. Reference temperature: 1st percentile of DMA-specific daily maximum heat index distribution.*

***Figure S9:*** *Time course for the relative risk of heat-related Google searches and 95% confidence interval for extreme high daily maximum heat index (95^th^ percentile of DMA-specific daily maximum heat index) with reference to the 1^st^ percentile of DMA-specific daily maximum heat index over lag 0-5 days. Note that the relative risk is displayed on the log scale.*

***Figure S10:*** *Cumulative exposure-response curve for the relative risk of heat-related ED visits for percentiles of DMA-specific daily maximum heat index over lags 0-5 days. Reference search volume: 1^st^ percentile of DMA-specific daily maximum heat index distribution.*

**

***Figure S11:*** *Time course for the relative risk of heat-related ED visits and 95% confidence interval for extreme high daily maximum HI (95^th^ percentile of DMA-specific daily maximum HI) with reference to the 1^st^ percentile of DMA-specific daily maximum HI over lag 0-5 days.*

***Table S6:*** *Relative Risk (95% confidence interval) for associations between daily maximum HI and searches at the 95th and 97.5th percentile of DMA-specific maximum HI; and HI and ED visits at the 95th and 97.5th percentile of DMA-specific maximum heat index. RRs are estimated with reference to the first percentile of searches or ED visits.*

| **Search Terms:** | **HI and Searches (95th percentile)** | **HI and Searches (97.5th percentile)** | **HI and ED visits (95^th^ percentile** | **HI and ED visits (97.5th percentile)** |
| --- | --- | --- | --- | --- |
| -- | -- | -- | 1.18 (1.13,1.22) | 1.19 (1.14,1.24) |
| **Air Conditioning** | 3.90 (3.27, 4.66) | 5.31 (4.24, 6.64) | -- | -- |
| **Air Conditioning (FBID)** | 8.27 (6.39, 10.69) | 12.06 (8.75, 16.62) | -- | -- |
| **Dehydration** | 1.46 (1.33, 1.61) | 1.54 (1.39, 1.71) | -- | -- |
| **Dehydration (FBID)** | 1.38 (1.26, 1.50) | 1.52 (1.36, 1.71) | -- | -- |
| **Heat** | 1.61 (1.50, 1.72) | 2.32 (2.00, 2.69) | -- | -- |
| **Heat (FBID)** | 1.64 (1.54, 1.75) | 2.44 (2.12, 2.76) | -- | -- |
| **Heat Exhaustion** | 22.17 (15.77, 31.18) | 33.70 (21.56, 52.69) | -- | -- |
| **Heat Rash** | 5.53 (4.27, 7.16) | 7.07 (5.15, 9.71) | -- | -- |
| **Heat Stroke** | 10.30 (8.12, 13.06) | 17.48 (12.27, 24.89) | -- | -- |
| **Swimming Pool** | 2.64 (2.30, 3.03) | 2.80 (2.39, 3.29) | -- | -- |
| **Swimming Pool (FBID)** | 3.33 (2.89, 3.83) | 3.43 (2.93, 4.01) | -- | -- |
